# Exposure of clownfish larvae to suspended sediment levels found on the Great Barrier Reef: Impacts on gill structure and microbiome

**DOI:** 10.1038/srep10561

**Published:** 2015-06-22

**Authors:** Sybille Hess, Amelia S. Wenger, Tracy D. Ainsworth, Jodie L. Rummer

**Affiliations:** 1 Australian Research Council Centre of Excellence for Coral Reef Studies, James Cook University, Townsville, Queensland, 4811, Australia; 2 College of Marine and Environmental Sciences, James Cook University, Townsville, Queensland, 4811, Australia

## Abstract

Worldwide, increasing coastal development has played a major role in shaping coral reef species assemblages, but the mechanisms underpinning distribution patterns remain poorly understood. Recent research demonstrated delayed development in larval fishes exposed to suspended sediment, highlighting the need to further understand the interaction between suspended sediment as a stressor and energetically costly activities such as growth and development that are essential to support biological fitness. We examined the gill morphology and the gill microbiome in clownfish larvae (*Amphiprion percula*) exposed to suspended sediment concentrations (using Australian bentonite) commonly found on the inshore Great Barrier Reef. The gills of larvae exposed to 45 mg L^−1^ of suspended sediment had excessive mucous discharge and growth of protective cell layers, resulting in a 56% thicker gill epithelium compared to fish from the control group. Further, we found a shift from ‘healthy’ to pathogenic bacterial communities on the gills, which could increase the disease susceptibility of larvae. The impact of suspended sediments on larval gills may represent an underlying mechanism behind the distribution patterns of fish assemblages. Our findings underscore the necessity for future coastal development to consider adverse effects of suspended sediments on fish recruitment, and consequently fish populations and ecosystem health.

Coastal development, dredging and other activities that result in suspended sediments in marine ecosystems are rapidly increasing^1^,^2^. For example, major coal and gas port expansions that are planned for within or adjacent to the Great Barrier Reef World Heritage Area will require dredging over 50 million cubic metres of sediment^2^. Suspended sediments are of global concern because of their association with seagrass loss and declines in coral diversity and abundance^3^. Reasons for the strong impact on corals include reduced light availability for photosynthesis, smothering of tissues, inhibition of larval settlement and an increased disease prevalence brought about by suspended sediments^4^,^5^,^6^. Recent evidence suggests that suspended sediments also negatively affect coral reef fishes, but the mechanisms underpinning these responses are not well understood.

Coral reef fish diversity and abundance has been noted to decline with increasing suspended sediment concentrations^5^,^7^,^8^. Partly, this has been attributed to loss of coral habitat^7^. However, direct effects of suspended sediments on coral reef fishes have been found, including the inhibition of visual and chemical cues important for foraging^9^, predator-prey interactions^10^, and habitat choice at settlement^11^. Previous research has also demonstrated that the pelagic stage of coral reef fish larvae is extended when fish are exposed to suspended sediment during development^12^. A disruption in larval development could significantly alter recruitment rates of coral reef fish to turbid reefs, thus shaping spatial distribution patterns^12^,^13^,^14^. Indeed, Srinivasan and Jones found that low recruitment rates of coral reef fish coincided with turbid water conditions associated with the summer monsoon in Papua New Guinea^15^. However, it remains unclear what mechanisms underpin the delay in pelagic larval development observed by Wenger and colleagues^12^.

Dispersing reef fish larvae have an extraordinarily high mass specific metabolic rate^15^, and therefore any reductions in oxygen uptake efficiency may compromise critical processes such as movement, growth and development^16^. Harmful effects of sediment particles on fish gills have been documented in freshwater and marine fishes^17^,^18^,^19^,^20^. However, responses seem to vary greatly between species, life stages, and sediment concentrations^18^,^19^. The gill epithelium consists of only a few cell layers in order to facilitate oxygen diffusion to the blood^21^. Morphological changes (e.g. mucous discharge or the growth of additional cell layers) at the gill epithelium can reduce the efficiency of oxygen uptake^21^,^22^. The gills can also be an entry point for pathogens into the body^23^, but the commensal bacteria communities on the gills can also act as first line of defence against pathogens^23^. Recently, suspended sediment exposure was linked to increased disease prevalence in coral colonies^6^, but studies examining the potential influence of suspended sediments on the microbiome of coral reef fish larvae, however, are lacking. The disturbance of the ‘healthy’ gill microbiome can facilitate the establishment and growth of pathogens on the gills and may thus increase the susceptibility of larvae to disease^23^,^24^.

The first goal of this study was to examine the effects of suspended sediment on the gill morphology of clownfish (*Amphiprion percula*) larvae using levels frequently found on inshore coral reefs of the Great Barrier Reef (GBR)^12^,^25^,^26^,^27^. The second goal of this study was to examine the effects of suspended sediment exposure on the gill microbiome of clownfish larvae. The Great Barrier Reef was used as a model system because of extensive literature on both suspended sediment concentrations^25^,^26^,^27^ and inshore to offshore gradients in species richness and abundance^5^. In addition, the larvae of many coral reef fishes on the GBR are exposed to relatively high levels of suspended sediments caused by flood plumes and re-suspension events (e.g. during storms) during their dispersal in the wet season^25^,^26^,^27^.

## Results

**Figure 1:**
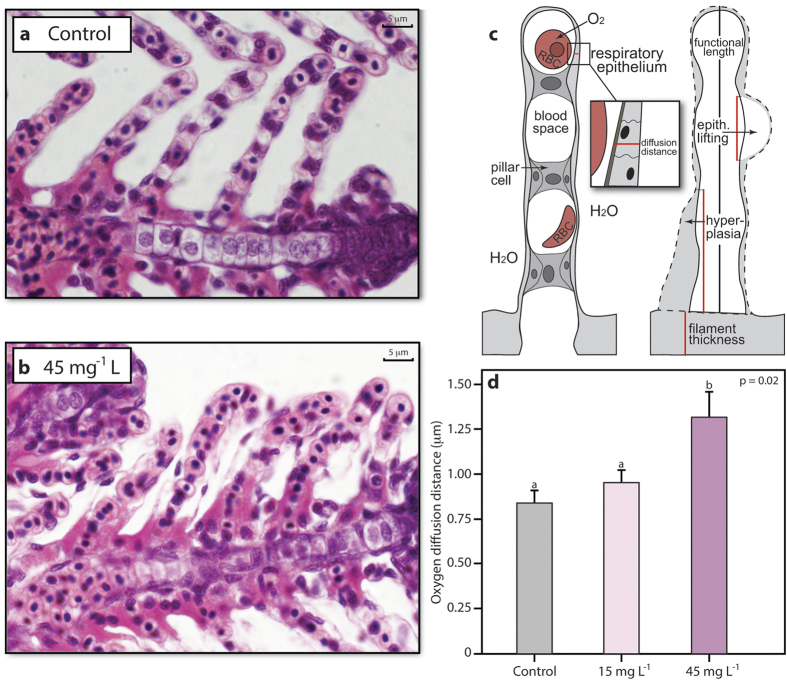
Gill morphology of fishes exposed to control conditions or suspended sediments. Representative micrographs of lamellae from a control fish (**a**) and a fish exposed to 45 mg L^−1^ suspended sediment concentrations with increased levels of hyperplasia (i.e. increased oxygen diffusion distances) at the respiratory epithelium (H&E stain) (**c**). Schematic of two lamellae depicting oxygen diffusion distance between water and blood (left) and gill changes (right). Red lines indicate distances measured. RBC: red blood cell, epith. lifting: epithelial lifting (**b**). Mean (±s.e.m.) oxygen diffusion distances at the respiratory epithelium between treatments (**d)**. Different lower case letters indicate significant differences between groups, using *P* < 0.05.

The gill morphology and the gill microbiome were significantly altered in clownfish larvae exposed to 45 mg L^−1^ suspended sediment concentrations (see Methods and Supplementary Methods) when compared to the control fish exposed to clean, filtered seawater. Mean oxygen diffusion distances (i.e., thickness of the gill epithelium) were 56% greater at the gills in fish exposed to suspended sediments than in control fish (Fig. 1a–d and Supplementary Table S1, *N* = 29, *t* = 2.32, *P* = 0.02). The increased diffusion distances resulted from additional cell layers accumulating on the gill epithelium (hyperplasia) (see also Supplementary Discussion). Low levels of hyperplasia can also be found in gills of healthy fish^28^. However, in the present study, hyperplasia covered significantly more (up to 68% more) of the gill lamellae in fish exposed to suspended sediment (37.6 ± 4.1% of lamellar length covered, mean ± s. e. m.) than in control fish (22.4 ± 2.6%) (Supplementary Fig. S1, *P* < 0.05). Fish that were exposed to 45 mg L^−1^ suspended sediment concentrations exhibited more than a doubling in mucous production at the gills (3.67 ± 0.7 μm^2^ mucous/μm of lamellar length) when compared to control fish (1.7 ± 0.37 μm^2^ mucous/μm of lamellar length) (Supplementary Table S1, *N* = 21, *t* = 2.10, *P* = 0.006). Individuals exposed to 15 mg L^−1^ were not examined. In three individuals, mucous completely congested all gill lamellae. Other aspects of gill morphology were unaffected by exposure to suspended sediment (see Fig. 1c and Supplementary Discussion). Furthermore, neither age nor standard length of the fish significantly influenced these analyses (Supplementary Table S1). We also examined the mucous cells on the skin, which is a secondary site for oxygen uptake during the larval stages of many fishes, including reef fishes^29^, and found approximately twice as many mucous cells on individuals exposed to suspended sediment (1.2 ± 0.2 cells / 100 μm skin) when compared to control fish (0.54 ± 0.1 cells / 100 μm) (see Supplementary Discussion, *P* < 0.01).

**Figure 2:**
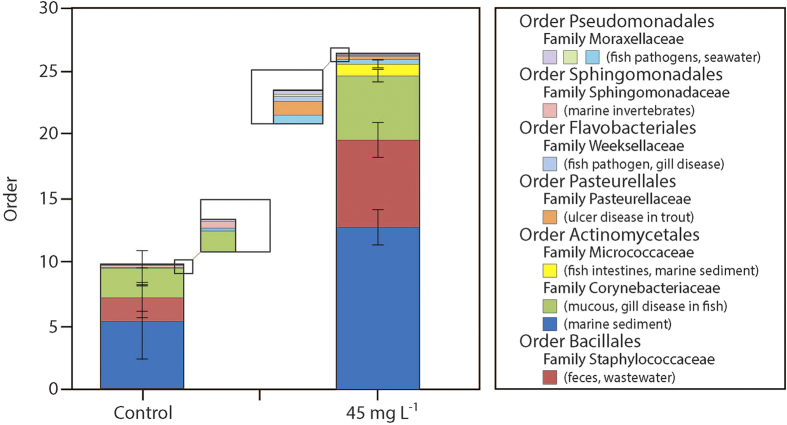
Bacteria with significant different abundances on gills of fish exposed to control conditions compared to gills of fish exposed to 45 mg L^−1^ suspended sediments. Potential pathogens and sources of bacteria are indicated in brackets. Statistical significance was assessed using *P* < 0.05.

Larval fish exposed to suspended sediments were also found to exhibit a different bacterial community to that of control fish. Out of the 93 616 DNA sequences retrieved from the larval fish heads (average OTU coverage 99.996% ± 0.113 (mean ± SEM)), 331 bacterial phylotypes (OTUs identified at >97% sequence homology) were identified in both treated and control fish, of which 140 where found exclusively in fish exposed to suspended sediments. Of particular interest are four OTUs that are members of groupings previously identified as teleost specific pathogens^23^ (*Flavobacterium*, *Pasteurella*, *Edwardsiella* and *Chryseobacterium* spp.) (Fig. 2 and Supplementary Table S2). In addition, two bacteria that are known to be associated with diseases specific to teleosts were isolated from both suspended sediment exposed and control fish but were found to be significantly more abundant in fish exposed to suspended sediment (*Pseudomonas* and *Corynebacterium* spp.; Fig. 2 and Supplementary Table S2, *P* < 0.05).

## Discussion

In the present study we demonstrate that exposure to suspended sediment concentrations frequently found on inshore reefs on the Great Barrier Reef negatively affects the gill morphology and the gill microbiome of clownfish larvae. Specifically, larvae exposed to 45 mg L^−1^ suspended sediment concentrations showed greater mucous discharge, a thicker gill epithelium and growth of various pathogenic bacteria on the gills when compared to control larvae.

The morphological changes at the gills observed in suspended sediment exposed larvae are assumed to protect the gill tissues (e.g., the pillar cell system) from abrasion from sediment particles^22^. Mucous discharge reduces the contact between sediment particles and the epithelium, while the growth of additional epithelial cell layers at the gills prevents the pillar cell system from rupturing^22^. These changes, however, increase the thickness of the epithelium and therefore increase the diffusion distance of oxygen between the water and the blood^21^. An increased diffusion distance can reduce the efficiency of oxygen uptake^21^, which can result in less oxygen being transported to organs than required, causing respiratory stress and reductions in performance^21^.

Respiratory stress can have negative consequences for all oxygen-consuming processes^16^,^30^. Dispersing reef fish larvae need to satisfy an extraordinarily high oxygen consumption rate in order to sustain rapid growth, development, and a high swimming activity shortly before settlement^15^,^16^. In a companion study, Wenger and colleagues examined the effects of suspended sediment exposure on growth, mortality, and development of larvae, and while they found no negative effects on larval growth or mortality when fish were exposed to 45 mg L^−1^ suspended sediment concentrations, larval development was significantly delayed^12^. Delayed development in response to respiratory stress has been described before in zebrafish^16^,^31^. Our findings suggest that the delayed development in larvae exposed to suspended sediments that Wenger and colleagues observed may have been a consequence of respiratory stress caused by changes in gill morphology. Further studies are required to examine the effects of suspended sediment exposure on oxygen uptake and the potentially negative ramifications on larval swimming activity and survival in the wild.

The bacterial community on larval gills in the present study changed significantly in response to suspended sediment exposure. In larvae exposed to suspended sediments, we found bacterial phylotypes in the same grouping as known culture identified bacterial pathogens. These phylotypes were not present in control fish, suggesting that transmission of pathogens can occur from suspended sediment to larvae^23^. In addition, potential pathogens present in only low abundances in control fish were found in significantly higher abundance in fish exposed to suspended sediments. These results may also indicate the presence of respiratory stress^32^ in suspended sediment exposed larval fish. Stress is known to facilitate the outbreak of diseases due to the suppression of the immune system^23^, and respiratory stress in particular has been shown to cause a shift from ‘healthy’ to pathogenic bacterial communities in salmon^32^. A proliferation of potential pathogens observed in suspended sediment exposed larvae may therefore be a consequence of the changes in gill morphology and resulting effects on oxygen uptake. The proliferation of pathogens facilitates disease outbreak^23^; however, we did not examine larvae for actual disease outbreak. Further studies are needed to examine the potential association between suspended sediment exposure and disease prevalence in fish larvae as well as the localisation of potential pathogens to key organs such as the gill.

Impaired gill function and increased susceptibility to diseases as a result of suspended sediment exposure may have important consequences for dispersal success of larvae in the wild^23^. Mortality of dispersing coral reef fish larvae is already extremely high, and even slight changes in mortality are known to have strong effects on adult populations^13^. The effect of suspended sediment exposure on the gills of clownfish larvae reported in the present study may represent a proximal mechanism contributing to changes in the structure of coral reef fish assemblages. The knowledge of such underlying mechanisms is crucial in order to predict how future levels of suspended sediments will affect fish populations, whether some species may be more vulnerable than others, and if acclimation and adaptation is possible.

Exposure of coral reefs to suspended sediments is rising, with at least 25% of coral reefs already threatened by poor water quality^33^. Worldwide, 275 million people reside within 30 km from coral reefs^33^, and some of the greatest increases in coastal development and population densities will be in emerging tropical economies, including those in the Coral Triangle – the global centre of coral reef biodiversity. Resources associated with coral reefs in the Coral Triangle currently sustain more than 100 million people^34^. Our results demonstrate that suspended sediments negatively impact fish larvae, suggesting that suspended sediments pose an even greater risk to coral reefs and the people who depend on them than previously understood. Suspended sediments caused by flood plumes and dredging activities can reach peak concentrations well above 500 mg L^−1^, which is a concentration more than eleven times greater than where we observed negative effects on larval fish^4^. Future development plans and dredging operations need to consider the potentially detrimental effects of suspended sediments on fish larvae and fish populations, which may require avoiding important fish habitats or larval dispersal seasons.

## Methods

### Experimental animals and larval rearing

For a full description of rearing conditions, see Wenger *et al.* 2014^12^. Experiments were conducted at the Marine and Aquaculture Research Facilities Unit (MARFU) at James Cook University, Townsville, Australia, between January and April, coinciding with the breeding season of *Amphiprion percula*. Each breeding pair was held in a 60 L outdoor aquarium supplied with filtered seawater (28 °C). Half of a terracotta pot was provided for egg deposition. The pots were checked for the presence of eggs each morning. If eggs were present, the pot was transferred to a temperature-controlled 60L indoor aquarium (28.5 °C) 6−8 days later, when hatching was predicted. Larvae were reared for five days in static conditions, and flushed gently with filtered seawater every morning. *Nannochoropsis spp*. paste (Reed Mariculture, California, USA) was added to tanks to dissipate light^35^. Diet of larvae consisted of rotifers (*Branchionus sp.*) for the first three days, and was then increasingly enriched with *Artemia sp*. nauplii (INVE technologies, Thailand LTD; GSL0).

### Experimental apparatus and experimental exposure

Larvae displayed sufficient swimming abilities to cope with the water flow present in experimental tanks from day 5 post-hatch^12^. On this day, larvae that were in good condition were distributed randomly to experimental tanks (at a density of 10 larvae per tank). 20 replicates (1 replicate = 1 tank) per treatment were conducted. A total of 24 2-L polyethylene terephthalate (PET) bottles were used as tanks, which were fitted with a 1 mm plastic mesh to prevent larval loss and shaded with black polyurethane sheeting. Larvae were then exposed to either 15 mg L^−1^ or 45 mg L^−1^ suspended sediment concentrations or clean, filtered seawater (control, 0 mg L^−1^). These suspended sediment concentrations are frequently found on inshore coral reefs of the Great Barrier Reef during the wet season^25^,^26^,^27^ (see also Wenger *et al.* 2014 and references therein). Each suspended sediment treatment was delivered via a submersible pump (1200 L h^−1^), which was situated in an external 100 L sump. Three disturbance pumps (1000 L h^−1^) in each sump kept the sediment suspended. The setting allowed for random allocation of treatments to tanks. All tanks were well-aerated, and dissolved oxygen levels were monitored daily (Cyberscan DO 300, Oakton Instruments, Vernon Hills, Illinois USA) to ensure they remained above 90% air saturation (5.6–6.2 mg L^−1^ at 28 °C). Turbidity in tanks (0 NTU in the control, ∼2.5 NTU in the 15 mg L^−1^ and ∼7.5 NTU in the 45 mg L^−1^ treatment) was measured with a WP88 Turbidity Meter, and found to be uniform. Australian Bentonite was used as the sediment, as it is a commercially available clay within the particle size range of natural sediments found on the Great Barrier Reef^12^,^36^,^37^. Larvae were checked for metamorphosis each morning before feeding. The appearance of the post-orbital stripe was used as a diagnostic tool for settlement^38^. Settlement was observed within 9 to 22 days after the start of the experiment. Once settled, larvae were removed from the tank and euthanized in a saltwater ice slurry.

### Histological preparation

Euthanized larvae were fixed in Bouin’s solution for 24 hours and then transferred to 70% ethanol. To measure standard body length, larvae were temporarily removed from the preservative and photographed on a 0.5 mm plastic grid. Because removing the gills was impractical due to the small size of larvae, whole larvae were serially dehydrated (Shandon Southern Duplex Processor BS5) and embedded in paraffin wax blocks (Shandon Histocentre 3, Thermo Electron Corporation). Fish were cut (5 μm, Micron HM 325) either longitudinally (control, 15, and 45 mg L^−1^ suspended sediment concentrations) and stained with Mayer’s hematoxylin and eosin, or dorso-ventrally (control and 45 mg L^−1^ suspended sediment concentrations) and stained with Periodic acid Schiff (PAS) and alcian blue (pH 2.5). Digital photographs (Olympus DP12 Microscope Digital Camera System) were taken at 1000x magnification to measure diffusion distance, width of the pillar cell system, and filament thickness. Hyperplasia, epithelial lifting, mucous cells, and mucous production at the gills were analyzed at 400x magnification. To count skin mucous cells, we analyzed two different dorso-ventral sections of the caudal fin and trunk.

### Gill morphology analyses

Measurements were carried out using ImageJ (Version 1.48, National Institutes of Health, USA), and blindly with respect to treatments. Developing lamellae at the tips of filaments were not analyzed. Measurements were taken as follows: *Oxygen diffusion distance*; the area of the functional lamella (i.e., area above the filament epithelium) was measured with the freehand selection tool. The area of the pillar cell system was also measured, subtracted from the area of the functional lamella, and then divided by twice the length of the functional lamella to obtain the oxygen diffusion distance. *Width of the pillar cell system*; the area of the pillar cell system was divided by the length to obtain the average width. *Thickness of the filament epithelium*; the filament thickness was measured starting at the base of the lamella (on each side of the lamella to calculate the mean). *Hyperplasia*; the length of lamellae covered by hyperplasia was measured (and % of lamellar length covered by hyperplasia was calculated). We chose to measure the lamellar length covered by hyperplasia because measuring the entire area covered by hyperplasia was not feasible. *Epithelial lifting*; same as for hyperplasia. *Mucous production of lamellae*; the area of the interlamellar space covered by mucous was determined by adjusting the color threshold of the micrograph^39^. To do so, the mucous area was roughly encircled with the freehand selection tool and copied into a new window. In the threshold color window (image → adjust → color threshold) white was selected as threshold color. Brightness, saturation and hue were adjusted until the area covered by mucous was white^39^. The area was selected and measured. *Mucous cells in the skin*; Individual micrographs of the caudal fin and the trunk of fish were fused together using the plugin ‘Stitching’ in ImageJ^40^. The mucous cells on the skin of the caudal fin and the trunk were counted, and divided by the length of the analyzed skin to account for size differences of larvae.

For the diffusion distance, the width of the pillar cell system, and the thickness of the filament epithelium, *N* = 10 fish and 204 lamellae in the control, *N* = 10 fish and 199 lamellae for the 15 mg L^−1^ and *N* = 9 fish and 180 lamellae in the 45 mg L^−1^ treatment were analyzed. For hyperplasia and epithelial lifting, *N* = 17 fish and 129 lamellae in the control, and *N* = 21 and 174 lamellae in the 45 mg L^−1^ treatment were used. For the number of mucous cells on the lamellae, *N *= 13 fish and 195 lamellae were used in the control and *N* = 13 fish and 225 lamellae in the 45 mg L^−1^ treatment. For the mucous production, *N* = 10 fish and 199 lamellae were used in the control, and *N* = 10 fish and 222 lamellae in the 45 mgL^−1^ treatment.

### PCR amplification of bacteria DNA, 454 tag sequencing and sequence analyses

The heads of fixed, paraffin-embedded larval fish were sampled following histological examination and exposure of the gill region within each larval head. The larval head was sampled using sterile 3 mm DNA/RNA free stainless steel cores to a depth of approximately 3 mm. Prior to coring, each paraffin block was first washed in DNA/RNA free water (3 × 5 min), molecular grade ethanol (3 × 5 min), and then held under UV for 40 min to clean and decontaminate the surface of the paraffin black. Each cored, paraffin-embedded fish head was then crushed, and the paraffin wax was removed by washing in molecular grade xylene (3 × 30 min), prior to washing in molecular grade ethanol (3 × 30 min at 37 °C) and DNAse/RNAse free water, digested in DNAse/RNAse free Proteinase K (50 mg ml^−1^) at 65 °C for 10 min, and further homogenized in a Fastprep at 4.5 m s^−1^ for 2 min prior to DNA extraction. DNA was extracted using a MoBio Powerplant PRO DNA kit (catalogue number 13400–50) following the manufacturer’s instructions. DNA extraction utilized previously unopened extraction kits for each set of samples, and negative controls were utilized at all amplification and sequencing stages (no contamination of extraction or sequencing buffers was identified). Following DNA extraction, samples were held at −20 °C prior to PCR amplification. Bacterial 16S rRNA genes were PCR amplified from the genomic template DNA in a single-step 30 cycle PCR (HotStarTaq Plus Master Mix Kit, Qiagen, Valencia, CA) under the following conditions: 94 °C for 3 min, followed by 28 cycles of 94 °C for 30 seconds; 53 °C for 40 seconds and 72 °C for 1 minute; after which a final elongation step at 72 °C for 5 min, using bacteria-specific barcoded primers 27F/519R with 454 A adaptor sequence.

Following PCR, all amplicon products from different samples were mixed in equal concentrations and purified using Agencourt Ampure beads (Agencourt Bioscience Corporation, MA, USA). Samples were sequenced utilizing Roche 454 FLX titanium instruments and reagents (MrDNA Texas) and negative controls for each of amplification and sequencing stage were utilized. The sequence data was processed using both a proprietary analysis pipeline (www.mrdnalab.com, MR DNA, Shallowater, TX) and the software package QIIME, where sequences were depleted of barcodes and primers then short sequences <200 bp are removed, sequences with ambiguous base calls removed, and sequences with homopolymer runs exceeding 6 bp removed. Sequences were then de-noised, chimeras removed (uchime utilising usearch5.2.236), and operational taxonomic units (OTUs) were defined at 97% similarity using QIIME. OTUs were then taxonomically classified using BLASTn against the curated GreenGenes database. Each of the samples was rarefied to 6200 sequences per sample to maximise the number of samples and sequences, allowing for 1 sample to be removed (Supplementary Fig. S3a). A Poisson embedding algorithm (Lladser, Gouet, & Reeder, 2011) was utilised to determine 95% confidence intervals for OTU coverage and to calculate the proportion of the community diversity captured via sequencing. Statistical analyses and data mining of bacterial data were conducted in QIIME and SPSS. Differences in community diversity (richness and Shannon index determined in QIIME) (Supplementary Fig. S3b,c) were compared using a Wilcoxon rank test and regression analysis. Significantly differentially abundant OTUs were identified using a Kruskal-Wallis rank test. OTUs found to be differentially abundant (*P* < 0.05) were subsequently classified based on highest sequence homology within BLASTn to amplicons obtained from aquatic environments.

### Statistical analysis of morphological data

Gill morphology analyses were performed in R (Version 3.0.2, R Core Development Team 2013). Linear mixed models (LMM) were used in the package lme4 to analyze oxygen diffusion distance, width of the pillar cell system, filament thickness, and mucous production. Treatment was entered into the models as the fixed effect, and age and standard length of fish were entered as covariates. Identity of each fish was included as a random factor to account for the use of multiple, non-independent measurements (i.e., lamellae) per fish (see Supplementary Information for equations). Assumptions of homoscedasticity and normality were checked by visual inspection of residuals and Q/Q-plots, and by Shapiro-Wilk tests. To meet assumptions, variables were log- or square-root transformed if necessary. Markov chain Monte Carlo (MCMC) sampling procedure was conducted to produce p-values. Length of hyperplasia, length of epithelial lifting and number of mucous cells on the gills were not analyzed using LMMs, however, as assumptions were not met, and Wilcoxon rank-sum tests were used instead. The measurements were averaged for each fish to avoid pseudo-replication. Number of mucous cells in the skin were analyzed with a Welch’s t-test after calculating the mean of the two sections per fish.

## Additional Information

**How to cite this article**: Hess, S. *et al.* Exposure of clownfish larvae to suspended sediment levels found on the Great Barrier Reef: Impacts on gill structure and microbiome. *Sci. Rep.* **5**, 10561; doi: 10.1038/srep10561 (2015).

## Supplementary Material

Supporting Information
