# Supplementary material for: Exposure of clownfish larvae to suspended sediment levels found on the Great Barrier Reef: Impacts on gill structure and microbiome
Source: Sci Rep. 2015 Jun 22;5:10561. doi: 10.1038/srep10561 (PMC5392994; doi:10.1038/srep10561)
Supplement: Supporting Information [file srep10561-s1.doc]

Supplementary Materials for

**Exposure to suspended sediment levels found on the Great Barrier Reef impacts gills and favours pathogenic bacteria in clownfish larvae**

Sybille Hess, Amelia S. Wenger, Tracy Ainsworth, Jodie L. Rummer

Correspondence to: amelia.wenger@my.jcu.edu.au

**This file includes:**

Figs. S1, S2, and S3
Tables S1 and S2
Supplementary Methods
Supplementary Discussion
Supplementary References

**Figure S1.** Percent of length of lamellae covered with hyperplasia in gills of fish exposed to the control or 45 mg L^-1^ suspended sediments. Asterisk indicates statistical significance between treatments, which was assessed using *P* < 0.05.

**Figure S2.** Box plot depicting number operational taxonomic units (OTU) of bacteria sequenced per gill core in fish from control (0 mg L^-1^ suspended sediments) and the 45 mg L^-1^ suspended sediment treatment. *N*= 6 fish per treatment.

**Figure S3.** Rarefaction curves with alpha diversity metrics for observed species (a), Chao1 index (b), and phylogenetic diversity (c) with average per sample for control (blue) and suspended sediment exposed (red) fish.

**Fig. S1.**

**Fig. S2.**

**Fig. S3.**

**
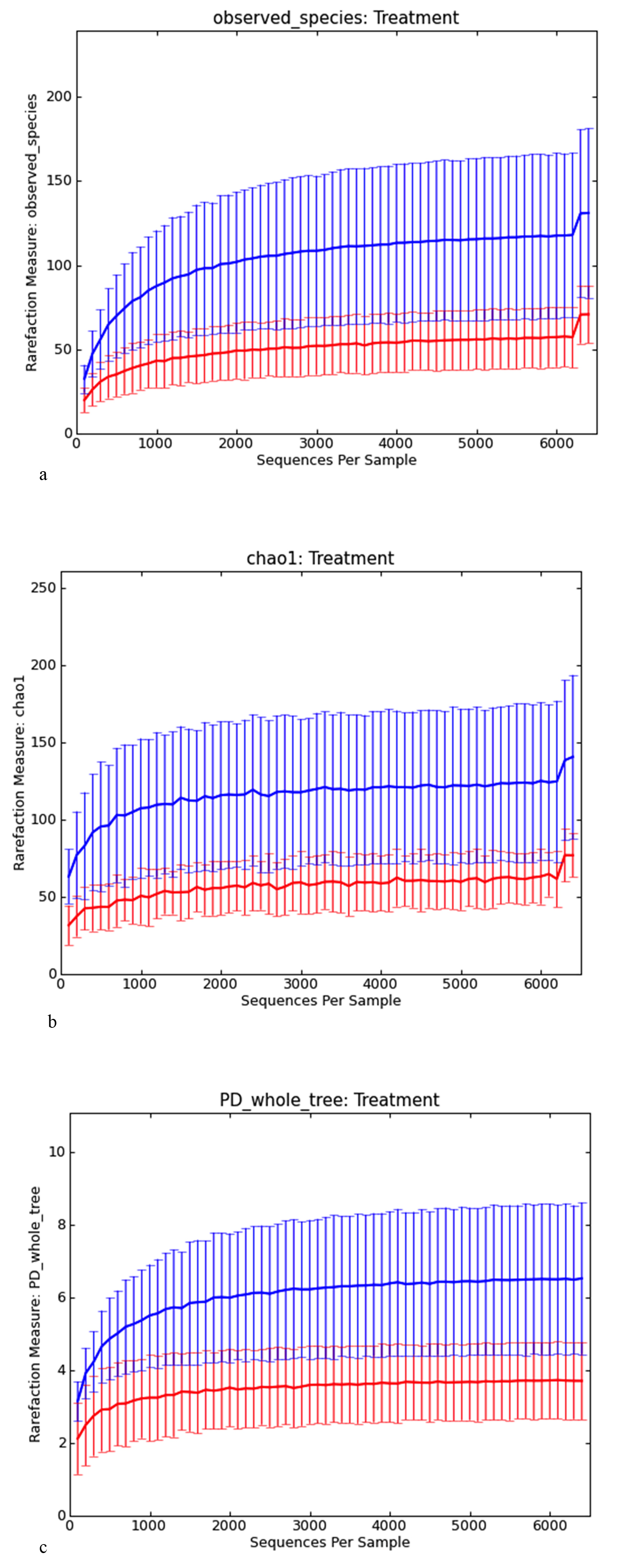
**

**Table S1.**

**Comparison of different aspects of gill morphology between treatments**. The control (0 mg L^-1^) was the reference category. Age and length of fish had no significant influence. *N* = 29 fish and 583 observations (i.e. lamellae) in a), b) and c), *N* = 21 fish and 421 observations in d). Significant factors are in bold (*P* < 0.05).

| Factors | Estimate ± SE | ***t*-value** | ***P*** | **Mean ± SEM** |
| --- | --- | --- | --- | --- |
| **a) Oxygen diffusion distance** | | | |  |
| Intercept | -0.29 ± 0.53 | -0.56 | 0.50 |  |
| Control |  |  |  | 0.84 ± 0.07 µm |
| 15 mg L^-1^ | 0.08 ± 0.13 | 0.61 | 0.48 | 0.95 ± 0.07 µm |
| 45 mg L^-1^ | 0.34 ± 0.15 | 2.32 | **0.02** | 1.32 ± 0.14 µm |
| Age | 0.03 ± 0.02 | 1.27 | 0.14 |  |
| Fish length | -0.02 ± 0.05 | -0.55 | 0.58 |  |
| **b) Width of pillar cell system** | | | |  |
| Intercept | 1.25 ± 0.20 | 6.12 | <0.0001 |  |
| Control |  |  |  | 3.09 ± 0.12 µm |
| 15 mg L^-1^ | -0.04 ± 0.05 | -0.85 | 0.29 | 2.96 ± 0.17 µm |
| 45 mg L^-1^ | -0.01 ± 0.05 | -0.20 | 0.81 | 3.01 ± 0.15 µm |
| Age | 0.00 ± 0.00 | -0.49 | 0.53 |  |
| Fish length | -0.00 ± 0.02 | -0.36 | 0.63 |  |
| **c) Thickness of filament epithelium** | | | |  |
| Intercept | 1.62 ± 0.24 | 13.91 | <0.0001 |  |
| Control |  |  |  | 3.52 ± 0.26 µm |
| 15 mg L^-1^ | -0.09 ± 0.06 | -1.67 | 0.10 | 2.96 ± 0.17 µm |
| 45 mg L^-1^ | -0.01 ± 0.06 | -0.21 | 0.80 | 3.67 ± 0.33 µm |
| Age | 0.01 ± 0.01 | 1.85 | 0.09 |  |
| Fish length | 0.00 ± 0.02 | 0.12 | 0.89 |  |
| **d) Mucous production** | |  |  |  |
| Intercept | 0.58 ± 1.44 | 0.40 | 0.58 |  |
| Control |  |  |  | 1.7 ± 0.37 µm |
| 45 mgL^-1^ | 1.53 ± 0.72 | 2.10 | **0.006** | 3.69 ± 0.7 µm |
| Age | 0.10 ± 0.12 | 0.81 | 0.41 |  |

**Table S2.**

**Potential pathogens found on gills of fish exposed to the control and 45 mg L^-1^ suspended sediments**. * Grouping linked to bacterial disease of fish; ** grouping linked to bacterial gill disease of fish and gill of fish; ^ found exclusively in treated fish; ^^ significantly different abundance in sediment exposed fish (*P* < 0.05).

| **OTU** | **Bacteria**  **(highest homology in aquatic sources)** | **Marine and aquatic sources** | **Homology** | **Accession number** | **Reference** |
| --- | --- | --- | --- | --- | --- |
| 2^^ | Actinomycetales,  *Tessaracoccus sp.*  *Arsenicicoccus sp.* | Sediment  Fish intestine | 89%  89% | NR_042550.1  NR_112991 | 42 |
| 4^^ | Bacillales,  *Bacillus sp.* | Faeces | 91% | NR_116886 | 43 |
| 5^^ | Actinomycetales, **Corynebacteriaceae^3^  *Corynebacterium sp.* | Mucus | 92% | NR_116647 | 45 |
| 13^ | Actinomycetales, Micrococcaceae  *Kocuria sp.* | Sediment | 95% | NR_118222  NR_025723 | 46  47 |
| 34^^ | Pseudomonadales, **Pseudomonas sp.*  *Marinomonas sp.* | Fish pathogen  Sediment | 89%  90% | NR_116899  NR_029228 | 48  49 |
| 44^ | Gammaproteobacteria  Pasteurellales,  **Edwardsiella sp.*  *Idiomarina sp.* | Fish pathogen  Seawater | 88%  89% | NR_1028131  NR_1168041 | 50  51 |
| 91^ | Flavobacteriales, **Flavobacteriaceae^3,11,12^  **Chryseobacterium sp.* | Fish pathogen | 85% | NR_0428261  NR_116480 | 54  55 |
| 139^^ | Sphingomonadales,  Sphingomonadaceae  *Sphingomonas sp.* | Marine invertebrate | 96%  94% | NR_041681  NR_041681 | 56  44 |
| 282^ | Pseudomonadales,  Acinetobacter  **Pseudomonas sp.*  *Marinomonas sp.* | Fish pathogen  Seawater | 92%  92% | NR_116899  NR_029228 | 48 |
| 349^^ | Pseudomonadales,  Acinetobacter  **Pseudomonas sp.*  *Marinomonas sp.* | Fish pathogen  Seawater | 91%  92% | NR_116899  NR_029228 | 48  49 |

**Supplementary Methods**

The following equations were used to assess the gill stress response in R.

(1) oxygen diffusion distance
lmer(log( (area.of.functional.lamella – area.of.pillar.cell.system)/(2*lamellar.length) + 1) ~ treatment + age + fish.length + (1fish.identity), data=data)

(2) width of pillar cell system
lmer(log(area.of.pillar.cell.system/ length.of.pillar.cell.system + 1) ~ treatment + age + fish.length + (1fish.identity), data=data)

(3) filament thickness
lmer(sqrt(filament.thickness) ~ treatment + age + fish.length + (1fish.identity), data=data)

(4) mucous production
lmer(area.covered.by.mucous/functional.lamellar.length ~ treatment + age + (1fish.identity), data=data)

**Supplementary Discussion**

Gills of fish larvae were still developing at the time of sampling. Most filaments carried 16 or less fully developed lamellae, and one or two lamellae that were differentiating at the top of the filament. Occasionally, a budding filament was observed on the gill arch, with no or only few short lamellae. Differentiating lamellae and budding filaments were excluded from analysis. The increased diffusion distance in the 45 mg L^-1^ suspended sediment treatment was due to a significant increase in lamellar hyperplasia (Wilcoxon rank-sum test, *N* = 38, *W* = 248, *P* < 0.05). In control fish, hyperplasia was mostly confined to the base of lamellae, growing in the “edges” between the filament epithelium and the lamella. 70% of lamellae in control fish showed this kind of hyperplasia, which covered less than 25% of the functional lamellar length. In contrast, in the 45 mg L^-1^ suspended sediment treatment, 60% of lamellae showed hyperplasia that affected more than 25% of the functional lamellar length, and covered both sides of the lamellae growing from the base towards the tip of lamellae. The tip of lamellae was usually not affected by hyperplasia.

Epithelial lifting covered 13.4 ± 3.9% of the functional lamellar length in control, and 11.2 ± 3.6% in 45mg L^-1^ suspended sediment in treatment fish, which was not statistically different (Wilcoxon rank-sum test, *N* = 38, *W* = 151.5, *P* = 0.43). The majority of lamellae (60%) in both treatments showed no epithelial lifting.

Mean number of mucous cells on lamellae was greater in fish of the 45 mg L^-1^ suspended sediment treatment (2.1 ± 0.6) than in control fish (1.2 ± 0.1), but this difference was not statistically significant (Wilcoxon rank-sum test, *N* = 26, *W* = 127, *P* = 0.18).

The increase in skin mucous cells in larvae exposed to 45 mg L^-1^ suspended sediment concentrations suggests that sediment particles not only had an influence on the gill epithelium, but may also have impacted the skin. The skin is a secondary site of oxygen uptake in fish larvae^29^, and future studies will be needed to examine if the effects of suspended sediments on the skin can impact cutaneous oxygen uptake. However, even if an increased mucous discharge on the skin did not have any negative effects on gas exchange, an increased mucous production requires additional energy, which adds to the already extremely high energy demand of dispersing reef fish larvae, and therefore would be expected to increase their risk of starvation^13, 17^.

**Supplementary References**

1. Lee, D. W. *Tessaracoccus flavescens sp*. nov., isolated from marine sediment. *Int. J. Syst. Evol. Microbiol.* **58**, 785 (2008).
2. Balcazar, J. L. *Bacillus galliciensis* *sp*. nov., isolated from faeces of wild seahorses (*Hippocampus guttulatus*). *Int. J. Syst. Evol. Microbiol.* **60**, 892 (2010).
3. Austin, B. & Austin, D. A. *Bacterial Fish Pathogens*. (Springer, Dordrecht, 2007).
4. Ben-Dov, E., Ben Yosef, D. Z., Pavlov, V. & Kushmaro, A. *Corynebacterium maris sp.* nov., a marine bacterium isolated from the mucus of the coral *Fungia granulosa*. *Int. J. Syst. Evol. Microbiol.* **59,** 2458–2463 (2009).
5. Bala, M., Kaur, C., Kaur, I., Khan, F. & Mayilraj, S. *Kocuria sediminis sp.* nov., isolated from a marine sediment sample. *Antonie Van Leeuwenhoek* **101,** 469–478 (2012).
6. Kim, S. B. *et al.* *Kocuria marina sp*. nov., a novel actinobacterium isolated from marine sediment. *Int. J. Syst. Evol. Microbiol.* **54,** 1617–1620 (2004).
7. Lopez, J. R. *et al.* *Pseudomonas baetica sp.* nov., a fish pathogen isolated from wedge sole, *Dicologlossa cuneata* (Moreau). *Int. J. Syst. Evol. Microbiol.* **62,** 874–882 (2012).
8. Solano, F. & Sanchez-Amat, A. Note: studies on the phylogenetic relationships of melanogenic marine bacteria: proposal of *Marinomonas mediterranea sp*. nov. *Int. J. Syst. Bacteriol.* **49,** 1241–1246 (1999).
9. Wang, Q. *et al.* Genome sequence of the versatile fish pathogen *Edwardsiella tarda* provides insights into its adaptation to broad host ranges and intracellular niches. *PLOS One* **4,** e7646 (2009).
10. Park, S. C. *et al.* *Pseudidiomarina aestuarii sp*. nov., a marine bacterium isolated from shallow coastal seawater. *Int. J. Syst. Evol. Microbiol.* **60,** 2071–2075 (2010).
11. Decostere, A., Haesebrouck, F., Turnbull, J. F. & Charlier, G. Influence of water quality and temperature on adhesion of high and low virulence *Flavobacterium columnare* strains to isolated gill arches. *J*. *Fish* *Dis*. **22**, 1-11 (1999).
12. Nematollahi, A., Decostere, A., Pasmans, F. & Haesebrouck, F. *Flavobacterium psychrophilum* infections in salmonid fish. *J*. *Fish* *Dis*. **26**, 563-574 (2003).
13. Bernardet, J. F. *et al.* Polyphasic study of *Chryseobacterium* strains isolated from diseased aquatic animals. *Syst. Appl. Microbiol.* **28,** 640–60 (2005).
14. Ilardi, P., Fernández, J. & Avendaño-Herrera, R. *Chryseobacterium piscicola sp*. nov., isolated from diseased salmonid fish. *Int. J. Syst. Evol. Microbiol.* **59,** 3001–3005 (2009).
15. Romanenko, L. A., Tanaka, N., Frolova, G. M. & Mikhailov, V. V. *Sphingomonas japonica sp*. nov., isolated from the marine crustacean *Paralithodes camtschatica*. *Int. J. Syst. Evol. Microbiol.* **59,** 1179–1182 (2009).
